# DLL3 serves as a pivotal hub bridging malignant progression and immunosuppression in NECC with therapeutic implications validated via the organoid-TIL co-culture system

**DOI:** 10.1016/j.gendis.2025.101737

**Published:** 2025-06-30

**Authors:** Qinqin Liu, Xinyu Qu, Shuqi Li, Yan Ding, Tingting Ren, Qi'an Jiang, Jingxin Ding, Keqin Hua, Junjun Qiu

**Affiliations:** a Department of Gynecology, Obstetrics and Gynecology Hospital of Fudan University, Shanghai 200090, China; b Shanghai Key Laboratory of Female Reproductive Endocrine-Related Diseases, Shanghai 200011, China

**Keywords:** Neuroendocrine carcinoma of the cervix, Organoid, Precise treatment, scRNA-seq, TCR-Seq, Tumor microenvironment

## Abstract

Neuroendocrine carcinoma of the cervix (NECC) represents a highly aggressive cervical cancer subtype with a poorly characterized tumor immune microenvironment (TIME) and unclear oncogenic mechanisms. Herein, through scRNA-seq analysis, we identified neuronal progenitor cells (NPCs) exhibiting high expression of NECC markers (SYN/INSM1) as putative malignant cells, and delineated three distinct NPC states along their developmental trajectory. Notably, we observed extensive infiltration of both naïve and exhausted T cells, collaboratively shaping an immunosuppressive TIME. Cellular communication analysis discovered DLL3's interaction with NOTCH2 on NPCs and NOTCH1/2 on tumor-infiltrating lymphocytes, with spatial relationships validated by multicolor immunohistochemistry staining. Functional experiments demonstrated that DLL3 promoted tumor cell proliferation and invasion, whereas co-culture assays revealed that DLL3 contributed to T cell exhaustion and impaired T cell activation, collectively proving DLL3 as a central molecular hub coordinating both malignant progression and immunosuppression. Moreover, we verified heightened and specific DLL3 expression in NECC tissues through immunohistochemistry staining, underscoring DLL3 as a promising therapeutic target for NECC. Significantly, we not only innovatively established NECC-derived organoids replicating cellular morphology, neuroendocrine differentiation features, and genetic characteristics of parental tumors, but also co-cultured organoids with tumor-infiltrating lymphocytes to assess the therapeutic efficacy of AMG757 targeting DLL3. Remarkably, the combination of AMG757 targeting DLL3 with chemotherapy (etoposide + cisplatin) induced substantial T cell aggregation and activation and heightened immunogenic cell death, offering novel insights into NECC treatment. Overall, the study provides comprehensive insights into NECC TIME and tailored therapeutic approaches beyond traditional therapy for NECC.

## Introduction

Despite widespread human papillomavirus vaccination and cytological screening, cervical cancer (CC) remains the fourth most common cancer and the fourth leading cause of cancer-related deaths in women.^1^ Neuroendocrine carcinoma of the cervix (NECC), a rare yet aggressive histological subtype, constitutes 0.9%–1.5% of invasive cervical cancers.^2^ Due to its propensity for early lymph node/vascular metastasis, most NECC patients are diagnosed at advanced stages during hysterectomy.^3^^,^^4^ Current multi-modality for NECC treatments, including surgery, chemotherapy, and radiation therapy, remains poorly effective,^4^ primarily extrapolated from squamous cell carcinoma (SCC)/adenocarcinoma of the cervix (ADC) or neuroendocrine carcinoma at other sites such as the lung and digestive tracts. Despite multi-modal therapy, 5-year survival rates for NECC remain poor (25%–39% across stages), deteriorating markedly in advanced stages.^2^^,^^5^ The persistent clinical challenges and unmet therapeutic needs in NECC underscore the imperative for systematic investigation into its biological behavior to elucidate the oncogenesis and inform therapeutic innovation.

The tumor immune microenvironment (TIME), pivotal in tumor development and prognosis, consists of diverse cellular/extracellular matrix components and signaling molecules that drive tumor growth and therapy resistance.^6^ Deciphering these complex interactions is essential for understanding cancer progression and overcoming therapeutic challenges.^6^^,^^7^ A research has indicated that neuroendocrine tumor cells derive from the embryonic neuroectoderm and exhibit immunohistochemical characteristics akin to those of endocrine gland cells,^2^ yet the precise etiology and origin of NECC remain elusive. Recent studies employing next-generation sequencing (NGS)^8^, ^9^, ^10^, ^11^ or whole exome sequencing (WES)^8^^,^^12^^,^^13^ have identified recurrent somatic mutations in NECC, delineating its distinct molecular profile and supporting the potential utility of targeted therapies. Moreover, integrated genomic-transcriptomic analyses reveal that NECC exhibits immune-excluded or “cold” tumor microenvironments compared to conventional cervical cancer.^14^ However, the TIME of NECC remains poorly characterized, and its oncogenic drivers are yet to be fully elucidated. This critical knowledge gap underscores the necessity to delineate the molecular mechanisms underlying immune evasion and tumorigenesis in NECC, which may identify novel therapeutic targets and guide precision immunotherapy strategies.

To elucidate malignant cellular origin, pathological aggressiveness, and actionable therapeutic targets of NECC, this study employs single-cell RNA sequencing (scRNA-seq) and T-cell receptor sequencing (TCR-seq) integrated with functional validation to systematically characterize the NECC TIME. By investigating neuronal progenitor cells (NPCs) and tumor-infiltrating lymphocytes (TILs; T and B cells), we seek to uncover the malignant origin and immune evasion mechanisms, which could also help identify novel treatment strategies. Notably, we revealed three states of synapsin (SYN) and insulinoma-associated protein 1 (INSM1)-expressing NPCs demonstrating functional plasticity, alongside the immunosuppressive TIME featuring massive naïve T cells and Tex cells infiltration, which collectively contributed to the marked aggressiveness of NECC. Additionaly, through cellular interaction analysis and functional assays, we identified delta-like ligand 3 (DLL3) as a pivotal molecular hub bridging tumor progression (proliferation/invasion) and immunosuppression (T cell exhaustion and impaired activation). Immunohistochemistry (IHC) staining confirmed DLL3's tumor-specific expression, highlighting its therapeutic potential in NECC. More importantly, we established an innovative *in* *vitro* 3D NECC-derived organoid-TIL co-culture system to evaluate the anti-tumor effects and T cell activation capacity of targeting DLL3 by AMG757, providing novel insights for NECC treatment.

## Methods and materials

### Patients and biospecimens

Our study was approved by the Ethics Committees of the Obstetrics and Gynecology Hospital of Fudan University (2022-105). We collected six tumor samples from two NECC, two ADC, and two SCC patients for scRNA-seq and TCR-seq. None of the patients received chemotherapy, radiotherapy, or any other anti-tumor therapy before surgery. We collected the blood sample from a healthy donor to collect T cells for co-cultivation with organoids. Another formalin-fixed samples were collected from the tissue bank of the Obstetrics and Gynecology Hospital of Fudan University (2020-22). All tissues were obtained with informed consent and with the approval of the ethics committees of our hospital. More detailed clinicopathological features of patients are listed in Supplementary Table S1.

### Preparation of single-cell suspension

The surgically removed tissues were kept in MACS Tissue Storage Solution (Miltenyi Biotec) until processing. The tissue samples were processed in the NovelBio's Laboratory according to the manufacturer's instructions. The samples were first washed with phosphate-buffered saline (PBS), minced into small pieces (approximately 1 mm^3^) on ice, and enzymatically digested with a tumor dissociation kit (Miltenyi Biotec) for 30 min at 37 °C with agitation. After digestion, the samples were sieved through a 70 μm cell strainer and centrifuged at 300 g for 5 min. After removing the supernatant, the pelleted cells were suspended in erythrocyte lysis buffer (Miltenyi Biotec) to lyse erythrocytes. After washing with PBS containing 0.04% bovine serum albumin (BSA), the cell pellet was resuspended in PBS containing 0.04% BSA and refiltered through a 35 μm cell strainer. The dissociated single cells were then stained with acridine orange/propidium iodide (AO/PI) for viability assessment using a Countstar Fluorescence Cell Analyzer. Following viability dye staining, cells were allocated for scRNA-seq.

### ScRNA-seq and TCR-seq

The scRNA-Seq libraries and V (D)J libraries were generated using the 10× Genomics Chromium Controller Instrument, the Chromium Single Cell 5′ library and gel bead kit, and the V (D)J enrichment kit (10× Genomics, Pleasanton, CA). The cells were concentrated to 1000 cells/uL and loaded into each channel to create single-cell Gel Bead-In-Emulsions (GEMs), resulting in 5000 single-cell expected mRNA barcodes per sample. After the reverse transcription step, GEMs were broken, and barcoded cDNA was purified and amplified. The amplified barcoded cDNA was used to construct 5′ gene expression libraries and TCR-enriched libraries. For 5’ library construction, the amplified barcoded cDNA was fragmented, A-tailed, ligated with adaptors, and index PCR amplified. For the V (D)J library, human T cell V (D)J sequences were enriched from amplified cDNA, followed by fragmentation, A-tailing, adaptor ligation, and index PCR amplification. The final libraries were quantified using the Qubit high-sensitivity DNA assay (Thermo Fisher Scientific), and the size distribution of the libraries was determined using a high-sensitivity DNA chip on a Bioanalyzer 2200 (Agilent). All libraries were sequenced on a 150 bp paired-end run by an Illumina sequencer (Illumina, San Diego, California, USA).

### ScRNA-seq data processing

The scRNA-seq data analysis was performed by NovelBio Co., Ltd. using the NovelBrain Cloud Analysis Platform (www.novelbrain.com). The raw data underwent quality control through filtering of adapter sequences and removal of low-quality reads using fastp with default parameters. The feature-barcode matrices were generated by aligning the reads to the human genome (GRCh38 Ensemble, version 100) using CellRanger v5.0.1. Downsample analyses were applied according to the mapped barcode reads per cell of each sample, and finally the aggregated matrix was achieved. Cells with over 200 expressed genes and a mitochondrial UMI rate below 20% passed the cell quality filtering, and mitochondrial genes were removed from the expression table. We used the Seurat package (version 3.1.4 https://satijalab.org/seurat/) for cell normalization and regression based on the expression table according to the UMI counts of each sample and percentage of mitochondrial rate to obtain the scaled data. Principal component analysis (PCA) was constructed based on the scaled data using the top 2000 highly variable genes, and the top 10 principal components were used for t-distributed stochastic neighbor embedding (t-SNE) and uniform manifold approximation and projection (UMAP) construction. Utilizing the graph-based clustering method, we acquired the unsupervised cell cluster result based on the PCA top 10 principal components. Marker genes were identified by the FindAllMarkers function with the Wilcox rank sum test algorithm under the following criteria: i) LnFC > 0.25; ii) *P*-value < 0.05; iii) min.pct > 0.1. To identify the detailed cell type, we performed re-tSNE/re-UMAP analysis, graph-based clustering, and marker analysis on clusters of the same cell type.

### Copy number variation (CNV) estimation based on scRNA-seq data

Myeloid and endothelial cells were defined as the reference to identify somatic CNVs using the R package infercnv (v0.8.2). We scored the extent of the CNV signal for each cell, which was defined as the squared mean of the CNV values across the genome. Putatively malignant cells were defined as those with a CNV signal > 0.05 and a CNV correlation > 0.5.

### GO analysis

We performed Gene Ontology (GO) analysis to elucidate the biological implications of marker genes and differentially expressed genes.^15^ We downloaded GO annotations from NCBI (http://www.ncbi.nlm.nih.gov/), UniProt (http://www.uniprot.org/), and the Gene Ontology (http://www.geneontology.org/). Fisher's exact test was applied to identify significant GO categories, and the false discovery rate was used to correct the *P*-values.

### KEGG pathway analysis

We performed pathway analysis to identify the significant pathways associated with marker genes and differentially expressed genes based on the Kyoto Encyclopedia of Genes and Genomes (KEGG) database. The Fisher's exact test was employed to determine the significant pathways, and the threshold of significance was established using *P*-value and false discovery rate.^16^

### QuSAGE analysis

QuSAGE analysis (2.16.1) was conducted to assess the relative activation of specific gene sets, such as “oxidative phosphorylation,” “cytokines,” “chemokines,” “immune response,” “cytotoxic,” “exhausted,” and “proliferative”.^17^

### Single-cell gene set enrichment analysis

Single-cell gene set enrichment analysis (ssGSEA), based on the domestic gene set and normalized gene expression matrix, was performed using the ssGSEA function in the GSVA package^18^ to obtain the gene enrichment score for each cell, including gene sets from the KEGG pathway database (https://www.kegg.jp/) and MSigDB (http://www.gseamsigdb.org/gsea/msigdb/index.jsp).

### Differential gene expression analysis

Differentially expressed genes among samples were identified using the FindMarkers function with the Wilcox rank sum test algorithm under the following criteria: i) lnFC > 0.25; ii) *P*-value < 0.05; iii) min.pct > 0.1.

### Pseudo-time analysis

Monocle2 (http://cole-trapnell-lab.github.io/monocle-release) was used for single-cell trajectory analysis with DDR-Tree and the default parameters. Marker genes from the Seurat clustering result and raw expression counts of the cells that passed filtering were selected before the Monocle analysis. Based on the pseudo-time analysis, we applied branch expression analysis modeling (BEAM analysis) for branch fate-determined gene analysis.

### Cell communication analysis

To enable a systematic analysis of cell–cell communication molecules, cell communication analysis was employed, utilizing the CellPhoneDB public database^19^ as a repository of information on ligands, receptors, and their interactions. The membrane, secreted, and peripheral proteins of the cluster at different time points were annotated, and significant means and cell communication significance (*P*-value < 0.05) were calculated based on the interaction and the normalized cell matrix achieved by Seurat normalization.

### TCGA public dataset analysis

Correlation analysis was conducted using the powerful online tool GEPIA (http://gepia.cancer-pku.cn/).^20^

### Multicolor immunohistochemistry staining

To investigate the multicellular ecosystem and spatial distributions of distinct cell types within TIME of NECC, we performed mIHC using formalin-fixed paraffin-embedded (FFPE) tissue sections from NECC, ADC, and SCC samples. The four-micron-thick FFPE tissue sections were stained sequentially with primary antibodies targeting platelet and endothelial cell adhesion molecule 1 (PECAM1) (ab182981, Abcam), cluster of diferentiation 38 (CD38) (ab108403, Abcam), CD8 (CST#70306S, CST), CD138 (ab128936, Abcam), and programmed cell death 1 (PDCD1) (CST#86163S, CST), along with a TSA 6-color kit (abs50015-100T, Absinbio). Additionally, the same tissue sections were also stained with primary antibodies targeting CD8 (CST#70306S, CST), SYN (ab32127, Abcam), PECAM1 (ab108403, Abcam), DLL3 (ab229902, Abcam), Notch1 (ab52627, Abcam), and Notch2 (5732T, CST), using the same TSA 6-color kit. Sections were then counterstained with 4′,6-diamidino-2-phenylindole (DAPI; abs47047616, Absinbio) to visualize nuclei. The antibodies used are listed in Supplementary Table S2.

To demonstrate the staining process, the PECAM1 signal was developed as follows. Deparaffinized sections were first incubated with anti-PECAM1 antibody for 30 min, followed by incubation with horseradish peroxidase-conjugated anti-rabbit secondary antibody (abs50015-02, Absinbio) for 10 min. The PECAM1 signal was then developed using TSA 520 for 10 min as per the manufacturer's protocol. Sections were then washed in Tris-buffered saline with Tween 20 (TBST) buffer and heated in preheated citrate solution (90 °C) using a microwave at 20% maximum power for 15 min. Sections were cooled to room temperature in the same solution. Between all steps, the sections were washed with Tris buffer. The same staining process was repeated for PECAM1/TSA 570, CD8/TSA 620, CD138/TSA 670, and PDCD1. Slides were air-dried and imaged using a Pannoramic MIDI II scanner (3DHISTECH). Images were analyzed with Indica Halo software.

### IHC analysis

Immunohistochemistry (IHC) procedures were conducted as described in our previous study.^21^ In brief, tumor tissue slides were rehydrated, subjected to antigen retrieval, blocked, and then incubated with primary antibodies. After 6 h, slides were treated with secondary antibodies and stained using a DAB peroxidase substrate kit for IHC (Yeasen, China). Three representative areas of each patient tissue were photographed (200× magnification), and the density of positive staining was quantified by measuring the integrated absorbance and image area. Based on the percentage of positive cells, the IHC staining area score was categorized as follows: 0 points (negative); 1 point (weak positive); 2 points (positive); 3 points (strong positive). Immunoreactive scores were calculated by multiplying the area and intensity scores. A list of the antibodies used in this study is provided in Supplementary Table S2.

### Cell culture

HeLa and NCI-H82 cells (ATCC) were maintained in Dulbecco's modified Eagle's medium (Gibco, 11965118) containing 10% FBS and 1% penicillin-streptomycin. Cells between passages 10–20 were used for experiments following authentication by STR profiling. Routine mycoplasma testing was performed using the MycoAlert Detection Kit (Lonza, LT07-218).

### Cell transfection

For cell transfection experiments, the DLL3-overexpression vector, DLL3-targeting knockdown vector, and their corresponding control vector (Genomeditech, Shanghai, China) were transfected into HeLa/NCI-H82 cells using Lipofectamine 2000 (11668-019, Invitrogen, Carlsbad, California, USA) in serum-free Opti-MEM medium (51985-034, Gibco, Gaithersburg, Maryland, USA).

### *In vitro* cytotoxicity assay

The cytotoxicity under different treatment conditions was assessed using the CCK8 assay. Organoids and TILs were seeded into culture plates and exposed to various drug treatments, including etoposide plus cisplatin (EP), AMG757, and their combination. Cell viability was evaluated by CCK-8 assay 72 h post-transfection. Cell viability was measured according to the manufacturer's instructions using the CCK8 assay, with absorbance recorded at 450 nm on a microplate reader. The viability of cells is calculated as [((As–Ab)/(Ac–Ab)] × 100% (As: treated groups; Ab: blank; Ac: control).

### RNA isolation, reverse transcription, and **quantitative real-time** analysis

Total RNA was isolated with Trizol reagent (Invitrogen, #15596026) and reverse transcribed using first-strand cDNA synthesis supermix (YEASEN, #11141ES60). Quantitative PCR was performed on Step One Plus Real-Time PCR System with SYBR green mix (YEASEN, #11203ES08), using β-actin as endogenous control. DLL3 expression was calculated by the 2^-ΔΔCt^ method with triplicate technical replicates.

### Western blotting

Proteins were extracted using either Western/IP lysis buffer or RIPA buffer containing protease/phosphatase inhibitors. Protein concentration was determined by bicinchoninic acid assay. Samples were separated by SDS-PAGE and transferred to PVDF membranes (0.45 μm) using a Bio-Rad system. After blocking with 5% BSA for 1 h, membranes were incubated with primary antibodies (4 °C, overnight) followed by secondary antibodies at room temperature for 1 h. Protein bands were visualized by enhanced chemiluminescence detection (ImageQuant LAS 4000) and quantified using ImageJ.

### Transwell assays

Cell migration and invasion were assessed using 8-μm pore Boyden chambers (Corning, #351152). Cells in serum-free medium were plated in the upper chamber, with 10% serum-containing RPMI 1640 as a chemoattractant. After incubation, migrated cells were fixed with 4% paraformaldehyde solution for 30 min, stained with crystal violet for 30 min, and then quantified by counting five random fields (Leica DMI3000 B).

### T cells expansion from peripheral blood mononuclear cells

Peripheral Blood Mononuclear Cells (PBMCs) were isolated from buffy coats with Ficoll–Paque according to the manufacturer's protocol (GE Healthcare). Culture media (“T cell medium”) for PBMCs was composed of RPMI 1640 (Gibco), supplemented with 2 mM ultraglutamine I, 1:100 penicillin/streptomycin and 10% male human AB serum (Sigma–Aldrich). One day before coculture, PBMCs were thawed in pre-warmed (37 °C) T cell medium (human serum was replaced with fetal calf serum during thawing) and incubated for 15 min with 25 U/mL benzonase (Merck). After washing, cells were resuspended at 2–3 × 10^6^ cells/mL in T cell medium supplemented with 150 U/mL interleukin-2 (IL-2) and cultured overnight at 37 °C.

### Generation of NECC-derived organoid-TIL co-culture system

The method of constructing NECC-derived organoids is as described previously.^22^ Cells were resuspended at a concentration of 2 × 10^6^ per mL in TIL cell culture medium supplemented with 150 IU/mL IL-2 and incubated overnight. The organoids were cultured overnight with 200 ng/mL interferon-gamma (IFN-γ) (PeproTech). A 24-well plate was coated with 5 μg/mL anti-CD28 (Biolegend) and incubated overnight at 4 °C. On the day of co-culture, organoids were dissociated into single cells using TrypLE Express (Gibco) and resuspended at a concentration of 5 × 10^4^ cells per mL in TIL cell culture medium. TILs (1 × 10^6^/well) and organoids were co-cultured at a 20:1 ratio in IL-2-supplemented medium for 3–5 days. All transfected cells underwent identical co-culture conditions as organoids (T cell ratio, duration, and IL-2 concentration).

### Flow cytometry

To detect intracellular proteins, cells were stimulated for 6 h with 10 μg/mL brefeldin A and 2 μm of ionomycin (Absin) before staining. Then, the cells were fixed and permeabilized using a fixation/permeabilization wash buffer (BioLegend) and stained with antibodies for 30 min in the dark. All the antibodies used in this study are listed in Supplementary Table S2. All samples were run on a CytoFLEX platform (Beckman Coulter) and analyzed using FlowJo version 10.8 software (BD Biosciences).

### Statistical analysis

All statistical analysis was conducted using Prism 8.0 (GraphPad Software). All graphs depict mean ± standard error of the mean unless otherwise indicated. Statistical significances are denoted as not significant (ns; *P* > 0.05), ∗*P* < 0.05, ∗∗*P* < 0.01, ∗∗∗*P* < 0.001, ∗∗∗∗*P* < 0.0001. The numbers of experiments are noted in the figure legends. The Mann–Whitney *U* test was used to compare two groups, and the Kruskal–Wallis test was used to compare multiple groups. Additionally, the Wilcoxon matched-pairs signed-rank test and the Friedman test were used for paired comparisons of two or multiple groups.

## Results

### Identification of unique Neuronal Progenitor Cells (NPCs) as the potential malignant origin of NECC

**Figure 1:**
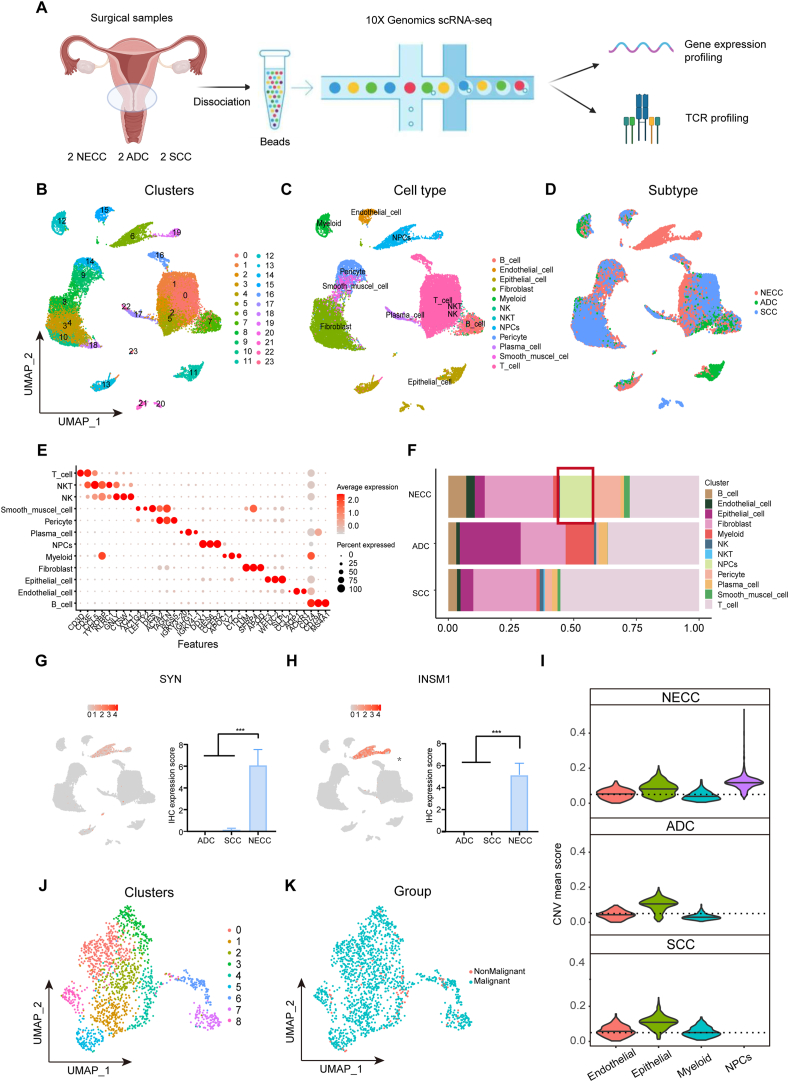
Identification of unique Neuronal Progenitor Cells (NPCs) as the potential malignant origin of NECC. **(A)** A schematic diagram illustrating the study design and analysis workflow. **(B**–**D)** A UMAP plot displays 24 clusters, 12 cell types, and 3 CC subtypes of 26,972 cells. Each dot represents a single cell, colored according to cell cluster, cell type, or patient origin. **(E)** Cell-type-specific marker gene expression in 12 cell types. Dot size indicates the proportion of expressing cells, colored by standardized expression levels. **(F)** The average proportion of distinct cell types in the NECC, ADC, and SCC groups, indicating remarkable intertumoral heterogeneity. The red box highlights the abundance of NPCs in NECC. **(G)** The left UMAP plot showing the SYN expression of NECC. The color keys ranging from gray to red indicate relative expression levels from low to high. The right boxplot showing the differences in immunohistochemical scores of SYN among 14 NECC, 9 ADC, and 14 SCC samples through IHC analysis. **(H)** The left UMAP plot showing the INSM1 expression of NECC. The color keys ranging from gray to red indicate relative expression levels from low to high. The right boxplot showing the differences in immunohistochemical scores of INSM1 among 12 NECC, 9 ADC, and 7 SCC samples through IHC analysis. **(I)** A violin plot showing CNV scores of endothelial cells, epithelial cells, myeloid cells, and NPCs in NECC, ADC, and SCC groups. **(J)** A UMAP plot exhibiting 9 subclusters generated via NPCs reclustering. **(K)** A UMAP plot showing malignant and nonmalignant NPCs determined using inferCNV analysis. Abbreviations: NPCs, neuronal progenitor cells; NECC, neuroendocrine carcinoma of the cervix; CC, cervical cancer; ADC, adenocarcinomas of the cervix; SCC, squamous cell carcinomas; CNV, copy number variation; IHC, immunohistochemistry. ∗, *p* < 0.05; ∗∗, *p* < 0.01; ∗∗∗, *p* < 0.001; ∗∗∗∗, *p* < 0.0001. ns, not significant; Error bar: mean value with 95% CI.

As the overall workflow shows (Fig. 1A), scRNA-seq and TCR-seq were performed on two NECC, two ADC, and two SCC samples to explore the unique characteristics of NECC distinct from SCC/ADC (Table S1). After quality control assessment, a total of 26,972 cells were subjected to dimensionality reduction, resulting in 24 clusters (Fig. 1B). According to canonical markers expression, we identified 12 distinct cell populations from cervical cancer samples (Fig. 1C–E), including T cells (CD3D, CD3E), natural killer T cells (CCL5, TYROBP, and KLRC1), natural killer cells (GNLY, CTSW, and XCL1), smooth muscle cells (ACTG2, LEFTY2, and DES), pericytes (ACTA2, TAGLN, and RGS5), plasma cells (IGKV3-20, IGHG1, and IGKV4-1), NPCs (DDX1, HES6, and CCER2), myeloid cells (APOC1, LYZ, and C1QC), fibroblasts (LUM, SFRP4, and APOD), epithelial cells (TFF3, WFDC2, and SLPI), endothelial cells (CCL21, AQP1, and ACKR1), and B cells (CD74, CD79A, and MS4A1). The abundance of each cell type varied considerably between NECC, ADC, and SCC (Fig. 1F). Remarkably, NPCs were found solely in NECC and demonstrated significant expression of SYN, a traditional diagnostic biomarker for NECC, as well as INSM1, a novel diagnostic biomarker for NECC recently recommended by the latest NCCN guidelines (Fig. 1G, H). Additionally, these findings were confirmed by IHC staining (Fig. S1A). Further inferCNV analysis revealed that NPCs in NECC, exhibiting extensive genomic variations, displayed higher CNV scores compared to control cells (endothelial and myeloid cells), suggesting their potential role as the malignant origin of NECC (Fig. 1I). In contrast, the malignant cells of SCC/ADC were epithelial cells, aligning with previous findings^23^ (Fig. 1I). Intriguingly, NPCs had even higher CNV scores compared to the malignant epithelial cells from ADC/SCC (Fig. S1B), indicating higher-grade aggressiveness of NECC compared to ADC/SCC. Subsequently, we reclustered NPCs from NECC and epithelial cells from ADC/SCC, and distinguished malignant cells from nonmalignant ones through inferCNV analysis (Fig. 1J, K; Fig. S1C, S1D). Specifically, malignant NPCs actively participated in the tumor necrosis factor (TNF) and mitogen-activated protein kinase (MAPK) signaling pathways, potentially mediating the oncogenic process, in contrast to the malignant epithelial cells derived from ADC and SCC, which were greatly involved in the cell cycle and viral carcinogenesis (Fig. S1E). These findings suggested NPCs featuring the expression of SYN and INSM1 as the malignant origin of NECC, implying the unique oncogenic mechanism of NECC different from ADC/SCC. Next, we would explore the features and functions of NPCs during NECC progression.

### Functional plasticity of three differentiated NPC states: edNPCs reshaping TIME, tdNPCs reprogramming cellular metabolism, and ldNPCs overactivating the cell cycle

**Figure 2:**
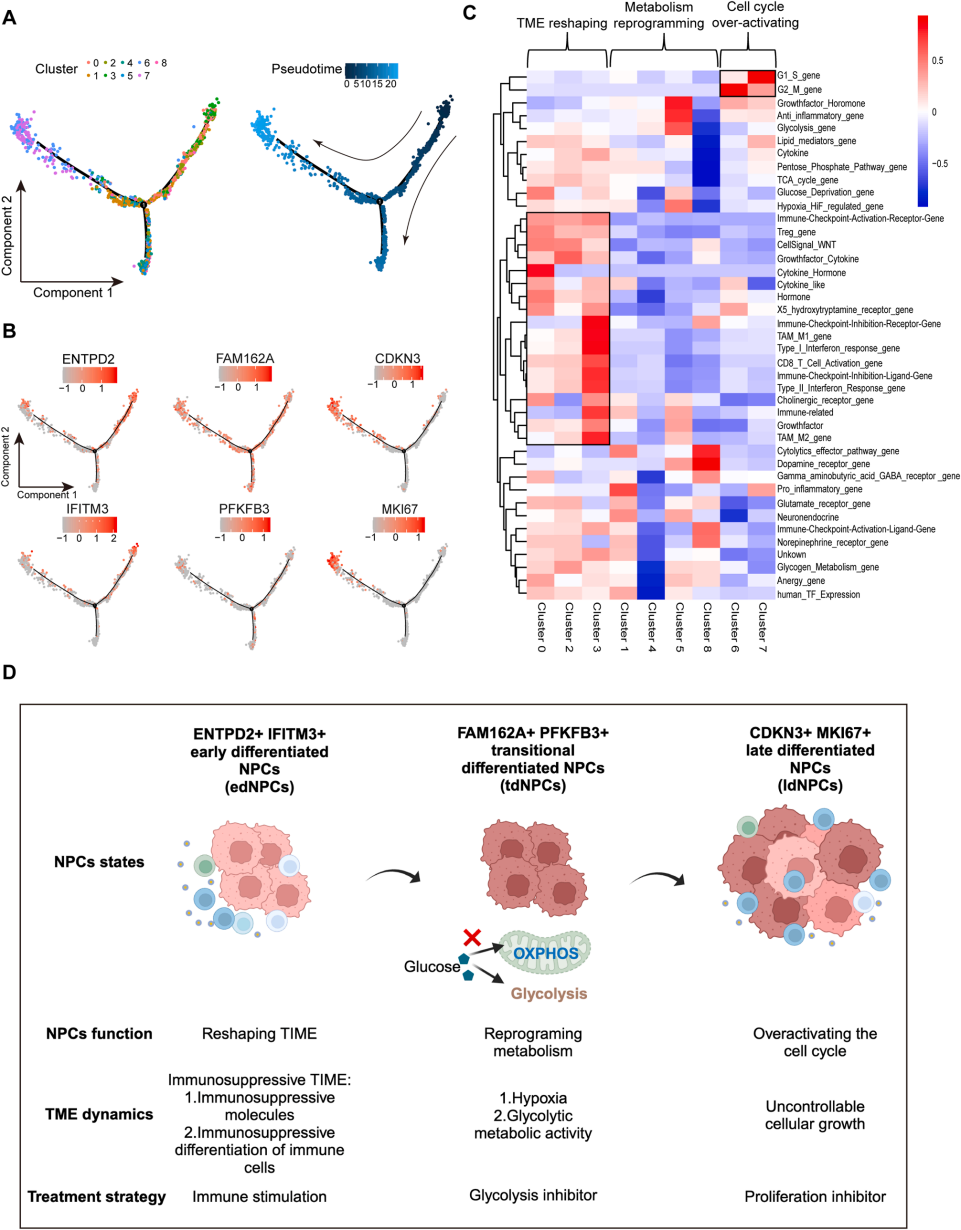
Functional plasticity of three differentiated NPC states: edNPCs reshaping TIME, tdNPCs reprogramming cellular metabolism, and ldNPCs overactivating the cell cycle. **(A)** Trajectory analysis of NPCs derived from NECC. NPC subclusters are labeled by colors. Shades from deep to light blue demonstrate the trajectory of cell differentiation and development: the darkest blue signifies an early differentiation stage, while the lightest blue represents a late differentiation stage. The arows indicate the differentiation and development trajectories of NPCs. **(B)** Dynamic expression of ENTPD2, IFITM3, FAM162A, PFKFB3, CDKN3, and MKI67 during the NPCs transitions based on pseudotime analysis. The color keys ranging from gray to red indicate relative expression levels from low to high. **(C)** Differentially expressed genes along the pseudo-time line were clustered hierarchically into three profiles (color key from blue to red). Representative gene functions and pathways are shown. **(D)** A schematic diagram showing the malignant progression of NPCs. Abbreviations: NPCs, neuronal progenitor cells; edNPCs, early differentiated neuronal progenitor cells; tdNPCs, transitional neuronal progenitor cells; ldNPCs, late differentiated neuronal progenitor cells; TIME, tumor immune microenvironment; NECC, neuroendocrine carcinoma of the cervix.

Trajectory analysis focusing on malignant NPCs was subsequently employed to trace the evolutionary pathway of NECC progression (Fig. 2A; Fig. S2A). Notably, NPCs in clusters 0, 2, and 3 congregated at the starting position, which were inferred to be “early differentiated NPCs” (edNPCs). Conversely, NPCs in clusters 6 and 7 were predominantly located at the end of the trajectory and were named as “late differentiated NPCs” (ldNPCs), whereas NPCs in clusters 1, 4, 5, and 8 were identified as “transitional differentiated NPCs” (tdNPCs). Noteworthy, edNPCs, characterized by the expression of ENTPD2 and IFITM3, were mainly involved in immune response-related pathways, such as type I/II interferon responses, cytokines, and immune checkpoint activation/inhibition receptors (Fig. 2B, C), potentially shaping the pro-tumoral inflammation and immune dysregulation. On the other hand, tdNPCs which exhibited high expression of FAM162A and PFKFB3, were related to hypoxia and glycolysis (Fig. 2B, C), demonstrating the dynamic change in the energy metabolism of NPCs. Additionally, ldNPCs, highly expressing CDKN3 and MKI67, were characterized by cell cycle-related pathways (G1_S and G2_M genes) (Fig. 2B, C), implying an increased proliferation capacity. Accordingly, we inferred that edNPCs and tdNPCs may potentially reshape the NECC TIME and reprogram cellular metabolism, providing a suitable soil and energy for the uncontrollable growth of ldNPCs, eventually manifesting as highly aggressive phenotypes of NECC. Moreover, the three differentiated NPCs also elicit precise therapeutic strategies for NECC treatment. Specifically, immunotherapy might enhance immune surveillance for edNPCs (ENTPD2^+^ IFITM3^+^), drugs targeting glycolysis might work for tdNPCs (FAM162A^+^ PFKFB3^+^), and anti-proliferative drugs, including platinum-based therapy and cyclin-dependent kinase (CDK) inhibitors, might effectively damage ldNPCs (CDKN3^+^ MKI67^+^).

In summary, we innovatively assigned three heterogeneous NPC types (ENTPD2^+^ IFITM3^+^ edNPCs reshaping TIME, FAM162A^+^ PFKFB3^+^ tdNPCs reprogramming cellular metabolism, and CDKN3^+^ MKI67^+^ ldNPCs over-activating the cell cycle) with functional plasticity, offering novel insights for NECC treatment (Fig. 2D).

### TILs (T and B cells) in NECC TIME: Inadequate immune activation and severe immunosuppression

**Figure 3:**
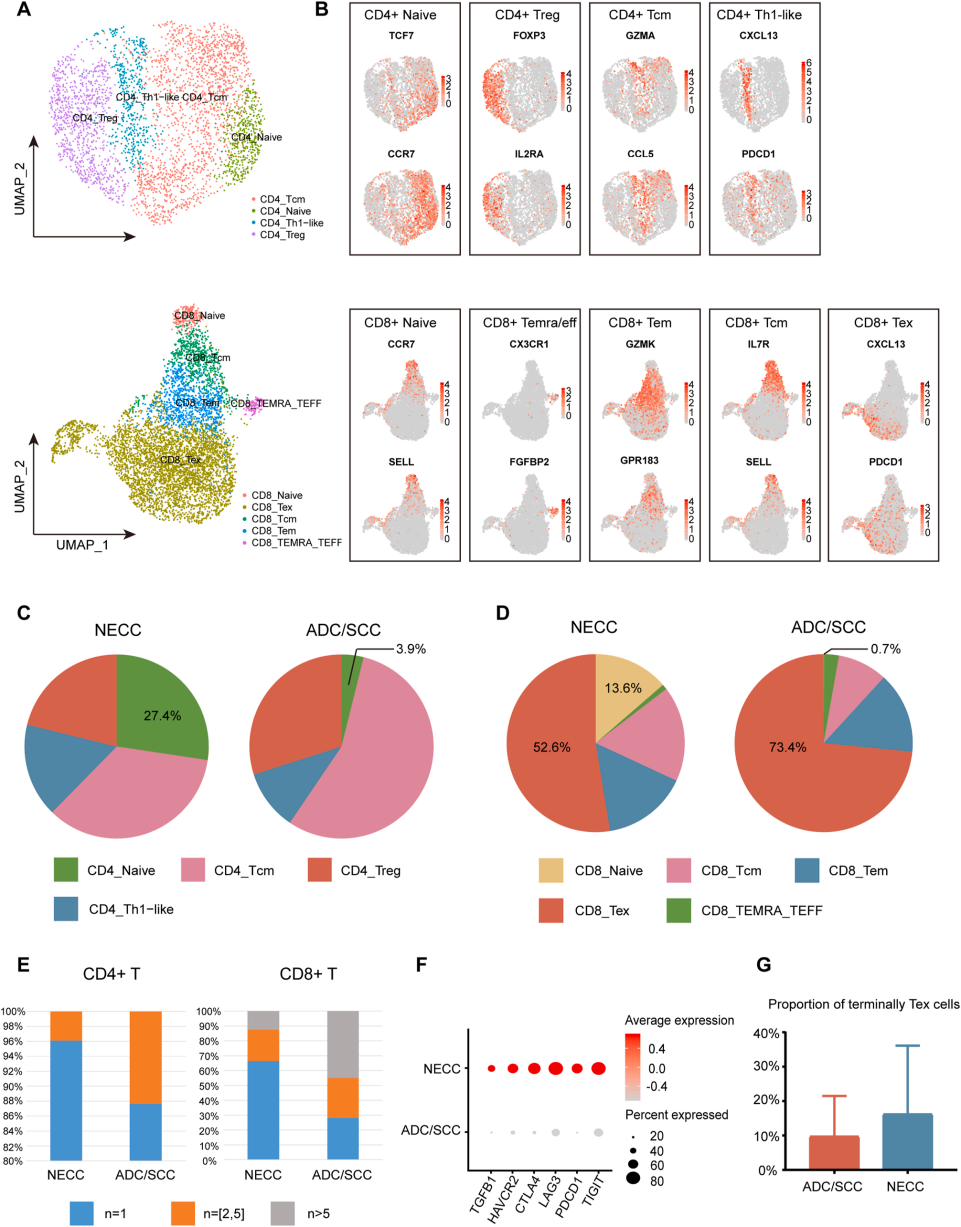
T cells in NECC TIME: Inadequate immune activation and severe immunosuppression. **(A)** UMAP plots showing the subgroups of CD4^+^ T cells and subgroups of CD8^+^ T cells derived from CC. **(B)** UMAP plots showing the normalized expression of common markers for subgroups of CD4^+^ T cells and subgroups of CD8^+^ T cells. The color keys ranging from gray to red indicate relative expression levels from low to high. **(C**, **D)** The average proportion of subtypes of CD4^+^ T cells (C) and CD8^+^ T cells (D) in the NECC and ADC/SCC groups. **(E)** A bar plot describing the TCR clonotype expansion in each CC sample. Left: CD4^+^ T cells; right: CD8^+^ T cells. **(F)** The expression of exhausted-related gene sets in Tex cells in each CC sample. The color keys ranging from gray to red indicate relative expression levels from low to high. **(G)** A histogram illustrating the proportion of terminally Tex cells (CD38^high^CD101^high^) within Tex cells in ADC/SCC and NECC. Abbreviations: NECC, neuroendocrine carcinoma of the cervix; TIME, tumor immune microenvironment; CC, cervical cancer; ADC, adenocarcinomas of the cervix; SCC, squamous cell carcinomas; TCR, T cell receptor; Tex cells, exhausted T cells.

Given that T cells predominated among immune cells in NECC (Fig. 1D), hinting at a pivotal role in NECC development and progression, we shifted our attention to the T cell profile of cervical cancer. Through unsupervised reclustering combined with cell annotation by classic markers, we identified four CD4^+^ T cell subgroups—regulatory T cells (Tregs) (FOXP3, IL2RA), Th1-like cells (CXCL13, PDCD1), Tcm cells (CCL5, GZMA), naïve T cells (CCR7, TCF7), and five CD8^+^ T cell subgroups—naïve T cells (CCR7, SELL), Temra/effector cells, Tcm cells (IL7R, SELL), Tem cells (GZMK, GPR183), and exhausted Tex cells (CXCL13, PDCD1) (Fig. 3A, B; Fig. S3A–S3D).

In NECC, we noted greater infiltration of CD4^+^/CD8^+^ naive T cells yet less infiltration of CD8^+^ Temra/effector cells compared to ADC/SCC (Fig. 3C, D). Besides, TCR-seq analysis revealed that T cells in NECC samples did not possess significantly higher expanded clonotypes than ADC/SCC samples (Fig. 3E). These findings suggested that a significant presence of naïve T cells within NECC TIME, without stimulation from tumor antigen signals, could weaken the anti-tumor response. Additionally, Tex cells within NECC exhibited higher exhaustion features (HAVCR2, CTLA4, LAG3, PDCD1, and TIGIT) compared to those in ADC/SCC (Fig. 3F), suggesting a more pronounced impairment in anti-tumor function of Tex cells in NECC compared to Tex cells in ADC/SCC. Fortunately, more than half of the Tex cells in NECC were found to be reversible Tex cells (CD38^low^CD101^low/high^ or CD38^high^CD101^low^) (Fig. 3G),^24^ suggesting potential reversibility despite compromised anti-tumor functionality. Such findings further highlighted the efficacy of immune checkpoint blocking therapy targeting specific immune checkpoint markers—HAVCR2, CTLA4, LAG3, PDCD1, and TIGIT—to restore T cell immune function, potentially benefiting NECC patients.

**Figure 4:**
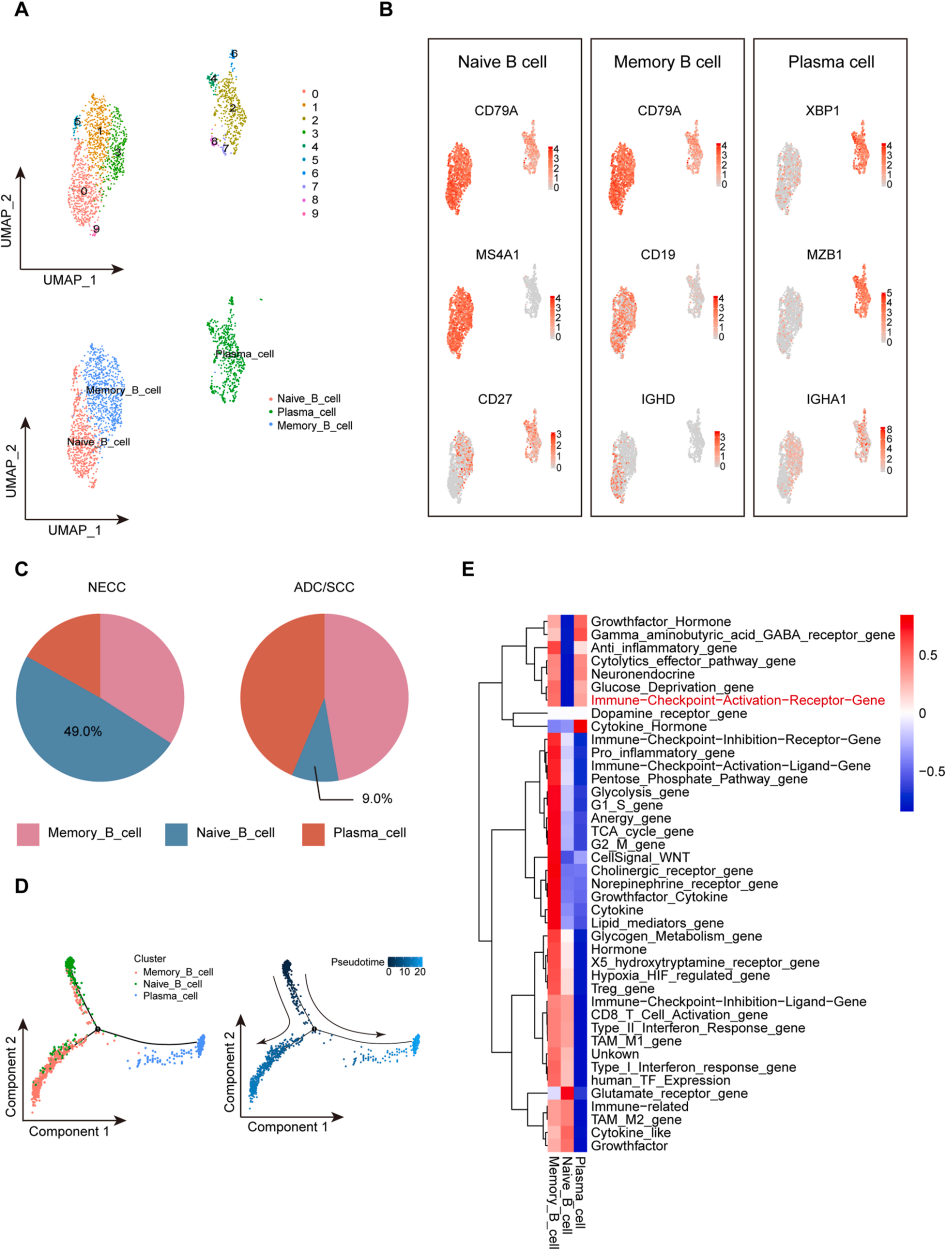
B cells in NECC TIME: Inadequate immune activation and severe immunosuppression. **(A)** UMAP plots showing the 10 clusters (upper panel) and three subgroups (lower panel) of B cells derived from CC. **(B)** UMAP plots showing normalized expression of common markers for B-cell subgroups. The color key from gray to red indicates relative expression levels from low to high. **(C)** The average proportion of B-cell subtypes in NECC and ADC/SCC groups. **(D)** Trajectory analysis of B cells derived from CC. Shades from deep to light blue demonstrate the trajectory of cell differentiation and development: the darkest blue signifies an early differentiation stage, while the lightest blue represents a late differentiation stage. The arrows indicate the differentiation and development trajectories of B-cell subgroups. **(E)** A heatmap showing the enriched functional pathways of B-cell subgroups with an enrichment score presented (color key from blue to red). Abbreviations: NECC, neuroendocrine carcinoma of the cervix; TIME, tumor immune microenvironment; CC, cervical cancer; ADC, adenocarcinomas of the cervix; SCC, squamous cell carcinoma.

Considering that B cells also play a crucial role in tumor immunity in addition to T cells,^25^ we further reclustered the B cells and identified three types of B-cell subgroups, including naïve B cells (CD79A, MS4A1, CD27), memory B cells (CD79A, CD19, IGHD), and plasma cells (MZB1, XBP1, IGHA1) (Fig. 4A, B). Compared to ADC/SCC, NECC had a higher proportion of naïve B cells (Fig. 4C), indicating the ineffective activation of B cells. Pseudo-time analysis revealed that naïve B cells subsequently differentiated into quiescent memory B cells and plasma cells (Fig. 4D), and the latter played a significant role in the activation of inhibitory immune checkpoints (Fig. 4E), thus facilitating the immune evasion of tumor cells.

Collectively, within NECC TIME, we observed a significant presence of inactive naïve TILs (T and B cells) and reversible exhausted T cells, highlighting the inadequate immune activation and severe immune suppression of TILs in NECC, further suggesting that activating naïve TILs and reversing exhausted T cells could be potential therapeutic strategies for NECC.

### Cellular communication analysis identifies DLL3 as a pivotal hub bridging malignant progression and immunosuppression

**Figure 5:**
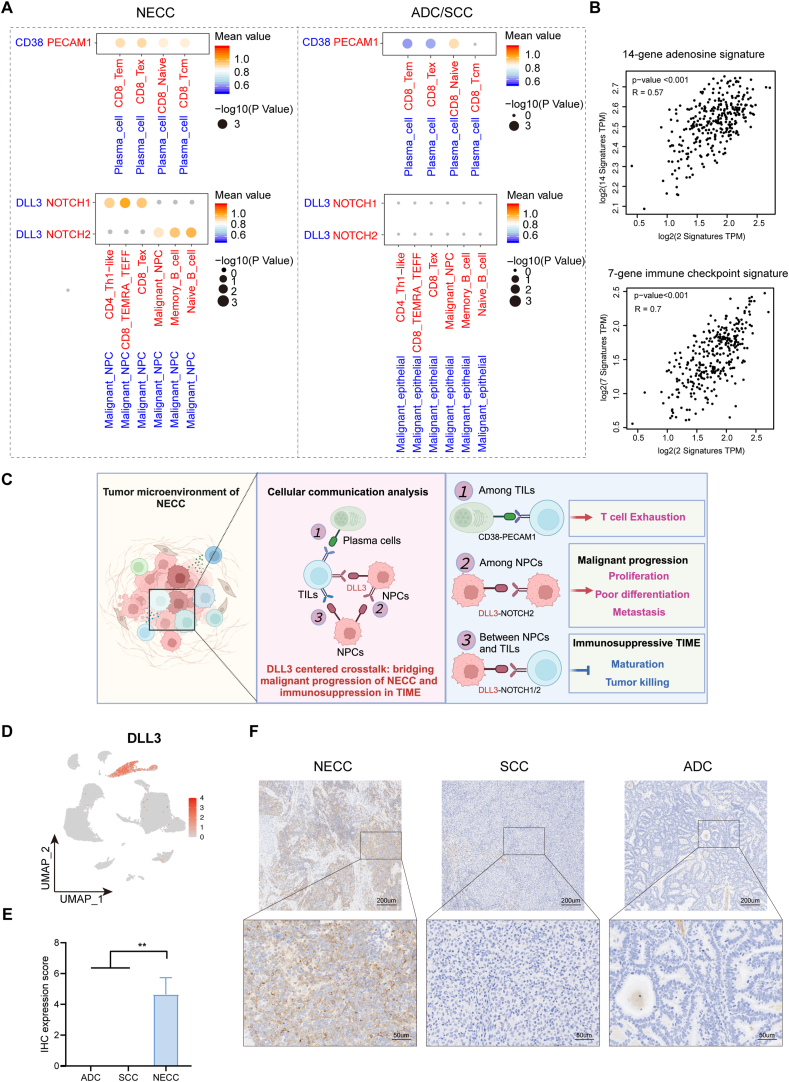
Cellular communication analysis identifies DLL3 as a pivotal hub bridging malignant progression and immunosuppression. **(A)** DLL3-NOTCH1/2 and CD38-PECAM1 interactions expressed in NECC and ADC/SCC samples. The cell subgroups and ligand–receptor interactions are indicated by the rows and columns, respectively. The means of the average expression levels of the two interacting molecules are indicated by the color key, with white to red representing low to high expression. The [−Log10(*p*-values)] are indicated by dot size. **(B)** Correlation between 2-gene signature (CD38, PECAM1) and 14-gene adenosine signature (PPARG, CYBB, COL3A1, FOXP3, LAG3, APP, CD81, GPI, PTGS2, CASP1, FOS, MAPK1, MAPK3, CREB1) or 7-gene immune checkpoint signatures (HAVCR2, TIGIT, LAG3, PDCD1, LAYN, CTLA4, TIM3) in CC at TCGA database. **(C)** Schematic diagram showing cellular communications (CD38-PECAM1, DLL3-NOTCH1/2) between NPCs and TILs in NECC. **(D)** A UMAP plot showing the expression of DLL3 of NECC. The color keys, ranging from gray to red, indicate relative expression levels from low to high. **(E)** The differences in immunohistochemical scores of DLL3 among 14 NECC, 9 ADC, and 7 SCC samples through IHC analysis. **(F)** DLL3 expression in representative NECC, ADC, and SCC samples by IHC staining. scale bars, 200 μm and 50 μm for the top and bottom panels, respectively. Abbreviations: NECC, neuroendocrine carcinoma of the cervix; TIME, tumor immune microenvironment; ADC, adenocarcinomas of the cervix; SCC, squamous cell carcinomas; TCGA, The Cancer Genome Atlas; NPCs, neuronal progenitor cells; TILs, tumor infiltrating lymphocytes; ∗, *p* < 0.05; ∗∗, *p* < 0.01; ∗∗∗, *p* < 0.001; ∗∗∗∗, *p* < 0.0001. ns, not significant; Error bar: mean value with 95% CI.

**Figure 6:**
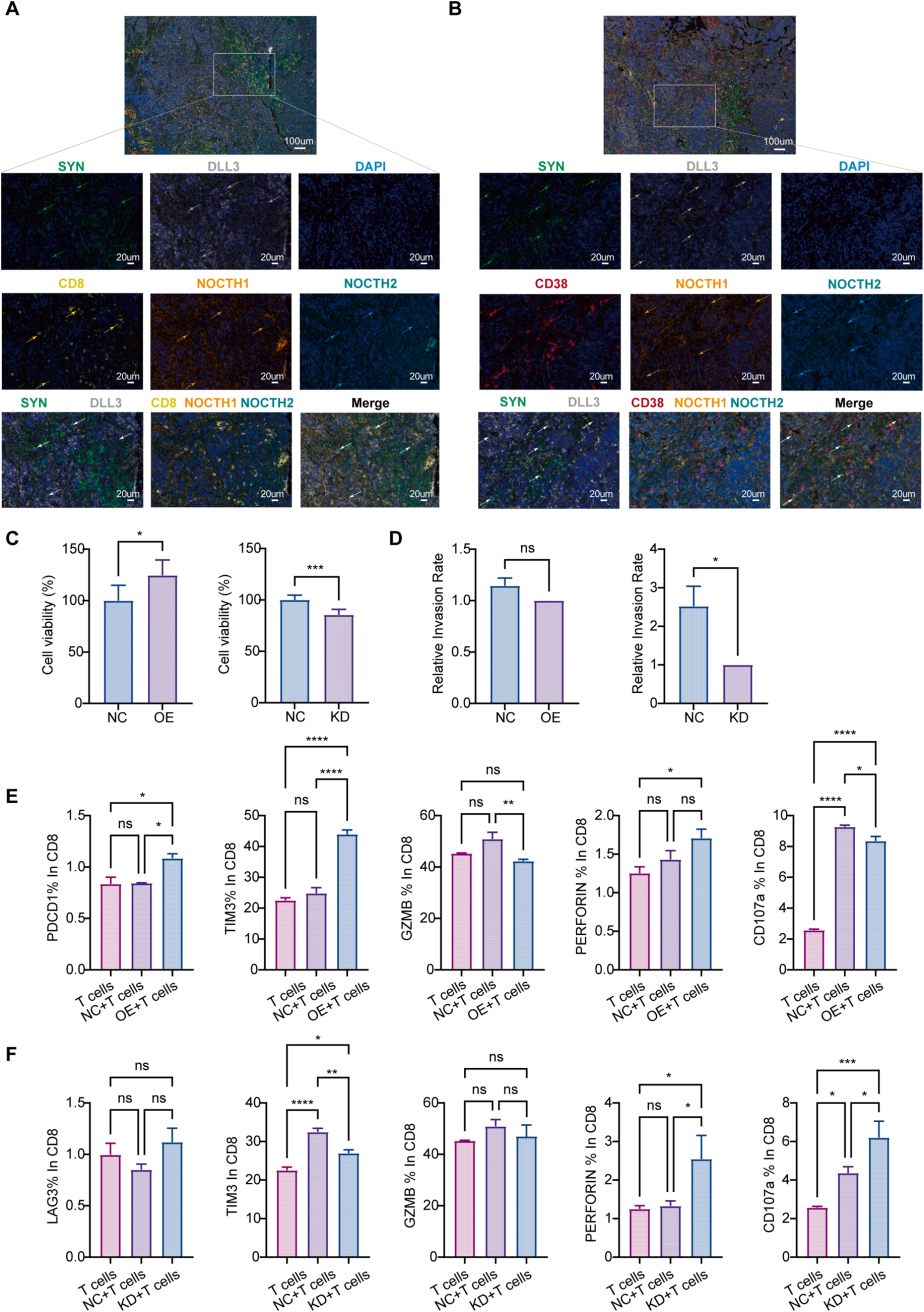
Functional assays identified DLL3 as a pivotal hub bridging malignant progression and immunosuppression. **(A)** mIHC staining showing the co-localization of DLL3^+^ tumor cells (white arrow) and NOTCH2^+^SYN ^+^ tumor cells (purple arrow) in NECC. The green, grey, yellow, orange and cyan-blue arrows indicate cells in NECC tissue samples with positive expression of the SYN, DLL3, CD8, NOTCH1, and NOTCH2 proteins, respectively. Scale bars, 100 μm and 20 μm for the top and bottom panels, respectively. **(B)** mIHC staining showing the co-localization of DLL3^+^SYN^+^ tumor cells (white arrow) and NOTCH1/2^+^ CD38^+^ B cells (purple arrow) in NECC. The green, grey, red, orange and cyan-blue arrows indicate cells in NECC tissue samples with positive expression of the SYN, DLL3, CD38, NOTCH1, and NOTCH2 proteins, respectively. Scale bars, 100 μm and 20 μm for the top and bottom panels, respectively. **(C)** HeLa DLL3 OE cells showed enhanced proliferation (left), while NCI-H82 KD cells exhibited reduced proliferation compared to their respective controls (right) (*n* ≥ 3 per group). **(D)** Invasion assays showing no statistically significant difference in invasive capacity between Hela-DLL3 OE and Hela-NC cells (left, *p* > 0.05, *n* ≥ 3 per group). NCI-H82-KD cells exhibited significantly reduced invasion compared to NCI-H82 NC cells (right, *p* < 0.05, *n* ≥ 3 per group). **(E)** The bar charts showing the expression of PDCD1, TIM3, GZMB, Perforin, and CD107a in CD8^+^ T cells co-cultured with HeLa NC/Hela-DLL3 OE cells through flow cytometry (*n* ≥ 3 per group). **(F)** The bar charts showing the expression of PDCD1, TIM3, GZMB, Perforin, and CD107a in CD8^+^ T cells co-cultured with NCI-H82 NC/NCI-H82 KD cells through flow cytometry (*n* ≥ 3 per group). Abbreviations: mIHC, multiplex immunohistochemistry; qRT-PCR, Quantitative Real-Time Polymerase Chain Reaction; OE, Overexpression; KD, Knockdown; NC, negative control. ∗, *p* < 0.05; ∗∗, *p* < 0.01; ∗∗∗, *p* < 0.001; ∗∗∗∗, *p* < 0.0001. ns, not significant; Error bar: mean value with 95% CI.

Having dissected the malignant NPCs and TILs (T and B cells) of NECC, we concentrated on the cellular interaction network within NECC TIME. Here are our key findings (Fig. 5A–C). (1) Among TILs within NECC, plasma cells interacted with various T cell subtypes through CD38-PECAM1 interaction. The expression of CD38 and PECAM1 were moderately correlated with the expression of a 14-gene adenosine signature (adenosine, an essential metabolic and immune checkpoint regulator promoting immunosuppression^26^) and 7-gene immune checkpoint signature based on the CESC cohort of the Cancer Genome Atlas (TCGA) database (Fig. 5B). Such findings manifested a nuanced role of plasma cells promoting the exhaustion of T cells through CD38-PECAM1 interaction, which was contrary to the conventional belief that plasma cells assist T cells against tumors. (2) Among NPCs within NECC, we noted that DLL3, uniquely expressed in malignant NPCs, interacted with NOTCH2 ligands in NPCs, which was reported to be associated with tumor progression and poor prognosis in several neuroendocrine neoplasms.^2^^,^^27^ These findings demonstrated a mutual influence between NPCs through DLL3-NOTCH2 interaction, promoting the malignant progression of NECC. (3) Among NPCs and TILs, we observed a high level of DLL3-NOTCH1/2 interaction between NPCs and various immune cells including memory B cells, Tex cells, CD4^+^ Th1-like cells, and Temra/effector cells, which has been reported to inhibit the maturation and anti-tumor effects of immune cells, thereby reshaping the immunosuppressive TIME.^28^ To summarize, we identified a DLL3-NOTCH1/2 signaling axis-mediated NPC-TIL interaction network in NECC, which may coordinately regulate tumor malignancy and immune evasion. Notably, immunohistochemical analysis indicated the specific expression of DLL3 in NECC samples (Fig. 5D), yet its complete absence in SCC and ADC cases (Fig. 5E, F). Further mIHC staining demonstrated co-localization of PECAM1^+^ CD8^+^ T cells and CD38^+^ CD138^+^ plasma cells (Fig. S4A), as well as co-expression of DLL3 and NOTCH2 in SYN^+^ tumor cells (Supplementary Fig. S4B). More importantly, we observed a distinctive spatial co-localization pattern of DLL3^+^ SYN ^+^ tumor cells and NOTCH1/2^+^ CD8^+^ T cells, as well as CD38^+^ B cells. Such characteristic cellular interaction was particularly prominent in neuroendocrine carcinoma cells (Fig. 6A, B) yet entirely absent in ADC/SCC cases (Supplementary Fig. S4C), highlighting the specificity of DLL3-NOTCH1/2 signaling axis in NECC.

Through comprehensive cell communication analysis supplemented with IHC/mIHC validation, we have demonstrated that DLL3, uniquely expressed in NPCs, might serve as a central molecular hub in NECC pathogenesis, simultaneously driving tumor progression through DLL3-NOTCH2 signaling and mediating immunosuppression via DLL3-NOTCH1/2 interactions with TILs.

### Functional validation of DLL3's multifaceted role in tumor progression and immunosuppression

To systematically investigate DLL3's dual roles in tumor progression and immune evasion, we established experimental models, including HeLa DLL3 OE (overexpression) and NCI-H82 (with neuroendocrine characteristics) DLL3 KD (knockdown), with transfection efficiency validated by quantitative real-time PCR and Western blotting (Fig. S3A–S3D). Of note, DLL3 overexpression significantly enhanced tumor cell proliferation (*P* < 0.05), while DLL3 knockdown markedly suppressed both proliferative capacity (*P* < 0.01) and invasive potential (Fig. 6C, D). Co-culture experiments with TILs demonstrated that DLL3-overexpressing cells induced potent immunosuppression, characterized by T cell exhaustion (PDCD1 and TIM3 upregulation) and impaired activation (GZMB and CD107a downregulation) (Fig. 6E), whereas DLL3 knockdown restored T cell cytotoxic function (Perforin and CD107a upregulation) and reversed exhaustion (TIM3 downregulation) (Fig. 6F). Intriguingly, treatment with the PDCD1 inhibitor toripalimab resulted in more tumor cell death in Hela-DLL3 OE groups compared to NC controls (*P* = 0.0543), while NCI-H82-DLL3 KD groups showed less tumor cell death than NC controls (*P* = 0.0922) (Fig. S4F). Although these differences did not reach statistical significance, the observed trends suggested that DLL3 might potentially serve as a predictive biomarker for immunotherapy efficacy in NECC, meriting further investigation. To summarize, the above findings position DLL3 as an attractive therapeutic target in NECC, offering the dual potential to arrest tumor progression and restore compromised immune reaction.

### The successful establishment of the NECC-derived organoid-TIL co-culture system to evaluate the efficacy of DLL3-targeted therapy

**Figure 7:**
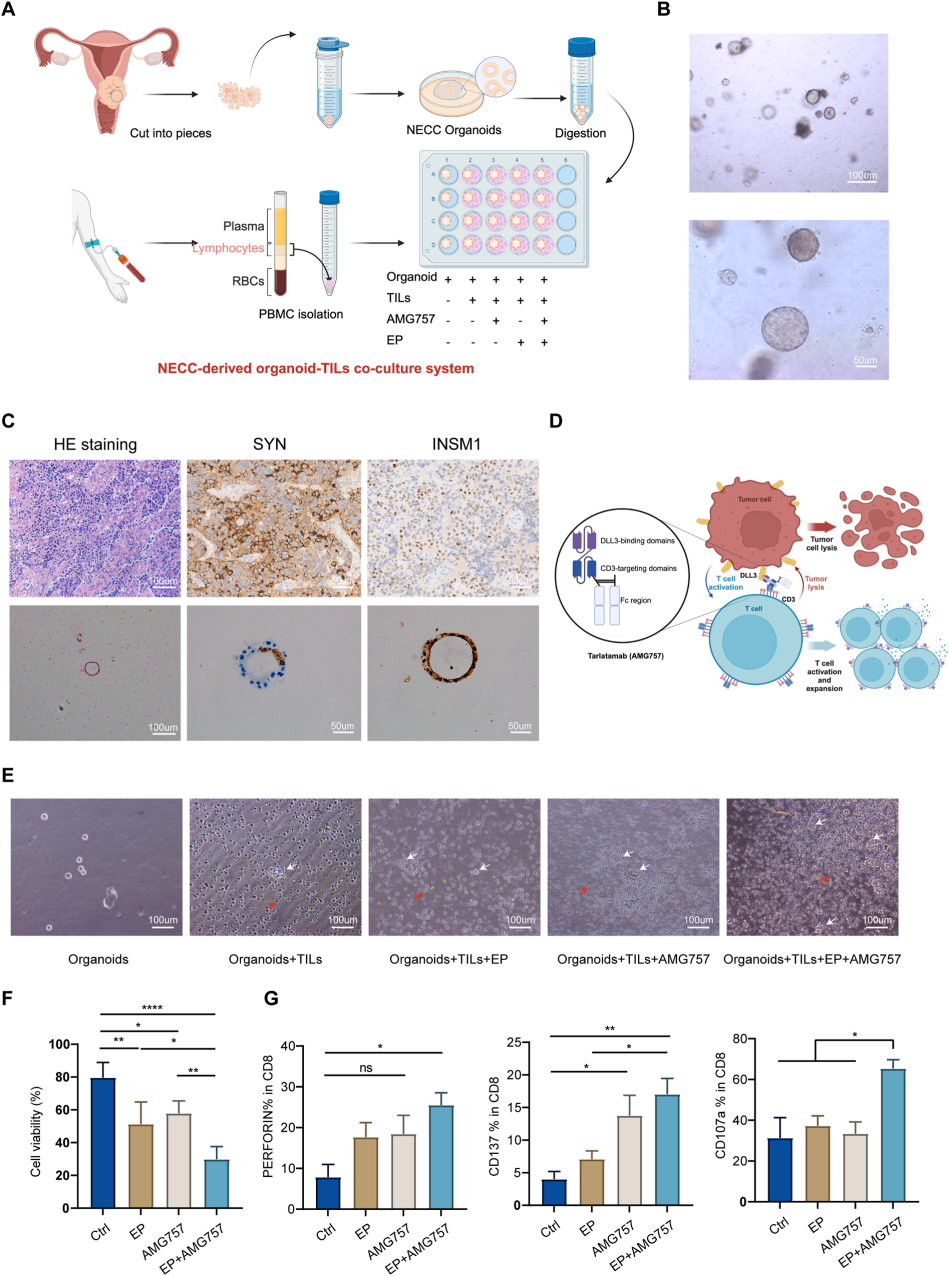
DLL3: A promising therapeutic target for NECC validated on NECC-derived organoid-TIL co-culture system. **(A)** Schematic diagram showing the establishment of NECC-derived organoid-TIL cell co-culture system. **(B)** Representative bright-field images of NECC-derived organoids remain viable *in vitro* for over 3 months. Scale bars, 100 μm and 50 μm. **(C)** Representative H&E staining and IHC staining (SYN, INSM1) of the parental NECC sample of the organoids. Scale bars: 100 μm and 50 μm. **(D)** Schematic diagram showing the treatment mechanism of DLL3-targeted therapy (AMG757). **(E)** Representative bright-field images of NECC-derived organoids and organoid-TIL co-culture system after various treatments. The red arrows indicate TILs, and the white arrows indicate NECC-derived organoids. Scale bars: 100 μm. **(F)** A bar chart showing the cell cytotoxic of different treatment regimens through CCK8 assays. **(G)** The bar charts showing the expression of PERFORIN, CD137, and CD107a in CD8+ T cells from NECC-derived organoid-TILs after different treatment regimens through flow cytometry (*n* = 4 for each treatment group). Abbreviations: NECC, neuroendocrine carcinoma of the cervix; TILs, tumor-infiltrating lymphocytes; H&E, Hematoxylin and eosin Staining; IHC, immunohistochemistry. ∗, *p* < 0.05; ∗∗, *p* < 0.01; ∗∗∗, *p* < 0.001; ∗∗∗∗, *p* < 0.0001. ns, not significant.

To further evaluate the efficacy of DLL3-targeted therapy, we established NECC-derived organoids viable for over three months under *ex vivo* conditions, given the scarcity of established NECC cell lines and animal models for experimental validation (Fig. 7A, B). We verified the consistency between the organoids and the original tumor tissue by the following means. 1) Hematoxylin-eosin staining manifested that NECC-derived organoids preserved the tissue architecture and cellular morphology of the original tumor tissue (Fig. 7C). 2) The organoids exhibited consistent neuroendocrine differentiation characteristics with the original tumor tissue, as evidenced by the expression of neuroendocrine markers (SYN, INSM1) (Fig. 7C). 3) WES analysis demonstrated a highly consistent mutation map between primary tumor tissue and NECC-derived organoids. 4) RNA sequencing of the organoids and their matched NECC samples revealed comparable gene expression profiles, indicating that transcriptome features were largely preserved in the NECC-derived organoids (Fig. S7A). Importantly, we also observed the consistant mutation presence of several frequently mutated genes (MUC16, MUC4) in the NECC-derived organoids, which further suggested the stable maintenance of the core mutation profile (Fig. S7B, S7C). Overall, these results demonstrated the successful establishment of a NECC-derived organoid model, which replicated the cellular morphology, neuroendocrine differentiation features, and genetic characteristics of the parental tumors, offering an *in* *vitro* platform to mimic NECC features. Since DLL3 might promote not only tumor proliferation and invasion but also immunosuppression in NECC TIME, solely establishing NECC organoids cannot precisely demonstrate the impact of anti-DLL3 on immune cells, especially on TILs. To address this issue, we proceeded to co-culture NECC organoids with TILs and created the NECC-derived organoid-TIL co-culture system (Fig. 7A), serving as a valuable *in vitro* tool to validate the efficacy of DLL3-targeted therapy.

### Targeting DLL3 with AMG757 exhibited optimal anti-tumor effects in the NECC organoid-TIL co-culture system

Using the organoid-TIL co-culture system, we evaluated the effect of DLL3-targeted therapy and its potential for enhancing the frontline chemotherapy regimen (etoposide + cisplatin, shortly named EP) for NECC. AMG757 is a DLL3-targeted bispecific T-cell engager that effectively binds to both DLL3 on cancer cells and CD3 on T cells to exert anti-tumor effects (Fig. 7D). Consistent with its expected mechanism of action, we observed a pronounced aggregation of T cells around NECC organoids in both groups treating with AMG757 and EP + AMG757 (Fig. 7E). Moreover, prominent cellular fragments were observed in the EP + AMG757 group indicating T cell-mediated tumor cells lysis (Fig. 7E). Further CCK8 experiments confirmed that the combination of EP and AMG757 demonstrated the most potent cytotoxic effect (Fig. 7F). According to flow cytometry analysis, we found that the EP + AMG757 therapy amplified the expression of co-stimulatory activation signal (CD137) and the release of cytotoxic granules (CD107a, Perforin) in CD8^+^ T cells compared to the other control groups, illustrating the activation of T cells (*P* < 0.05) (Fig. 7G). Overall, these findings suggested that EP combined with AMG757 could induce T cell activation and promote immunogenic cell death, providing broader treatment avenues for NECC patients, particularly for those with positive DLL3 expression.

## Discussion

**Figure 8:**
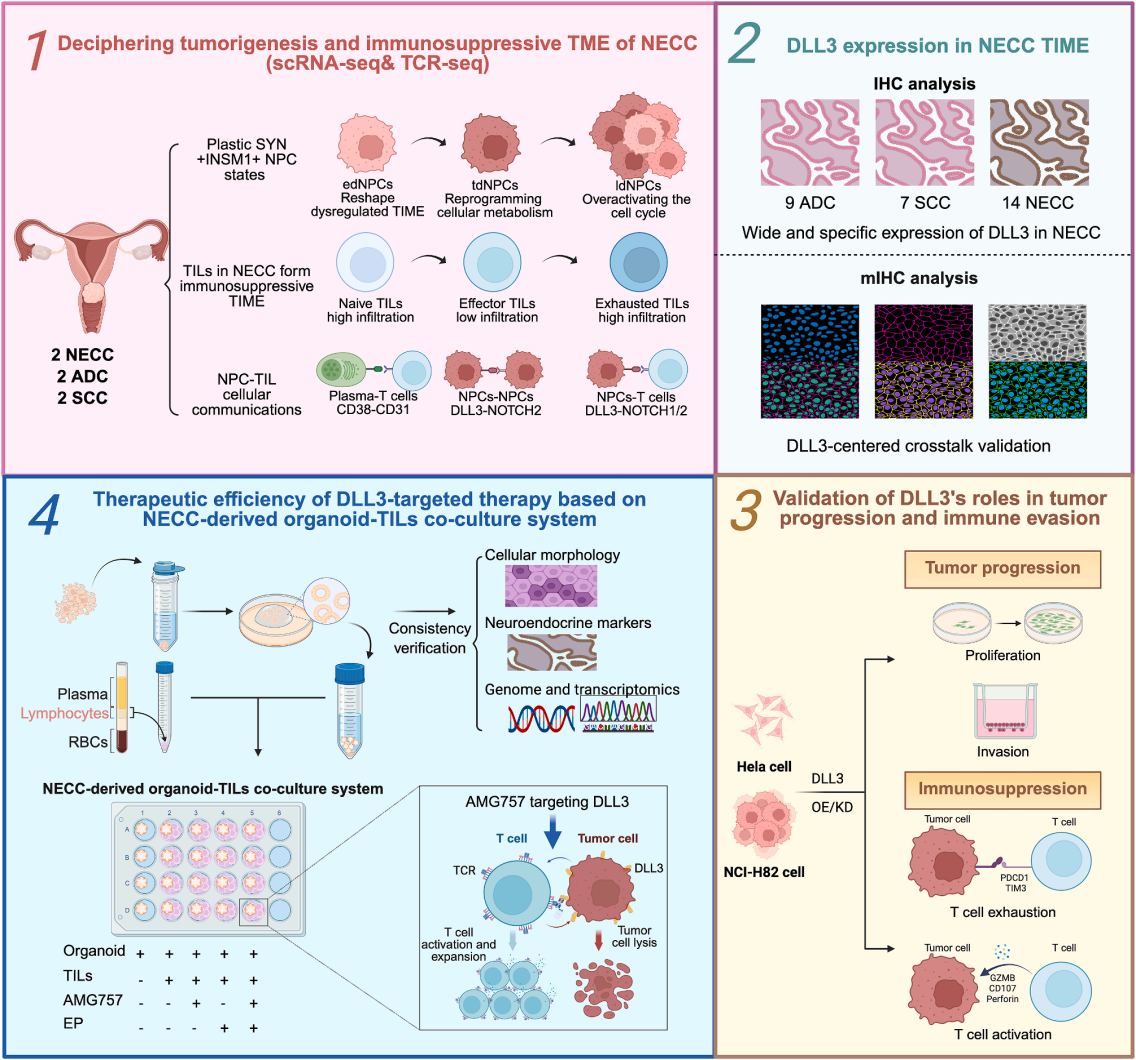
Graphical abstract of the workflow of the study and significant findings. Abbreviations: TME, tumor microenvironment; NECC, neuroendocrine carcinoma of the cervix; scRNA-seq, single cell RNA sequencing; TCR-seq, T cell receptor sequencing; NPCs, neuronal progenitor cells; TILs, tumor infiltrating lymphocytes; IHC, immunohistochemistry; mIHC, multiplex immunohistochemistry; ADC, adenocarcinomas of the cervix; SCC, squamous cell carcinomas; EP, etoposide + cisplatin; OE, overexpression; KD, knockdown.

NECC represents a rare and highly aggressive histological variant of cervical cancer, characterized by its distinctive “4U” features: uncertain origin, ultra-aggressive behavior, unsatisfactory treatment outcomes, and unavailability of experimental models. To address these challenges, we innovatively mapped the single-cell transcriptomic landscape of NECC and revealed its unique oncogenic mechanism distinct from ADC/SCC, which was mainly attributed to the three states of SYN and INSM1-expressing NPCs, and the immunosuppressive TIME featuring extensive infiltration of naïve and exhausted TILs. Through cellular communication analysis and functional validation assays, we found that DLL3 emerged as a pivotal molecular hub bridging malignant progression and immunosuppression in NECC TIME. Additionally, IHC staining verified the high and specific expression of DLL3 in NECC, highlighting DLL3 as a promising therapeutic target. Considering the limited cell line and animal models of NECC, we successfully established NECC-derived organoids replicating cellular morphology and neuroendocrine differentiation characteristics of parental tumors, and further co-cultured the organoids with TILs to evaluate the effect of DLL3-targeted therapy (AMG757) and its potential for enhancing the frontline chemotherapy regimen (EP) for NECC. Encouragingly, AMG757 combined with chemotherapy triggered substantial T cell activation and increased immunogenic cell death, suggesting the great potential for clinical use of DLL3 targeting. Briefly, our study not only unveiled SYN and INSM1-expressing NPCs as the malignant origin of NECC and highlighted abundant infiltration of naïve T cells and Tex cells in NECC TIME, but also validated DLL3-targeted therapy as a promising strategy for NECC patients via the organoid-TIL co-culture system (Fig. 8).

To date, the exact oncogenesis of NECC from the perspective of TME has not been clearly elucidated. In this study, we identified NPCs featuring the expression of SYN and INSM1 as the malignant origin of NECC. Moreover, we discovered three differentiated NPC types (ENTPD2^+^ FITM3^+^ edNPCs reshaping TIME, FAM162A^+^ PFKFB3^+^ tdNPCs reprogramming cellular metabolism, and CDKN3^+^ MKI67^+^ ldNPCs over-activating the cell cycle), which elicited specific treatment strategies including immune activation, glycolysis inhibitors, and anti-proliferative drugs targeting the corresponding differentiated states. Additionally, our results indicated that NECC was infiltrated with a large number of Tex cells, highlighting an immunosuppressive TIME in NECC. Fortunately, due to the abundant reversible Tex cells (CD38^low^ CD101^low/high^ or CD38^high^CD101^low^) in NECC that highly expressed immune checkpoint targets such as HAVCR2, CTLA4, LAG3, PDCD1, and TIGIT, immune checkpoint blocking therapy may hold significant clinical value in restoring exhausted immune cells against NPCs, requiring further preclinical investigation. In summary, we uncovered three-differentiated NPCs with functional plasticity and immunosuppressive TIME with extensive infiltration of naïve and reversible exhausted TILs, providing crucial clues for NECC treatment options.

Current multi-modality treatments for NECC, including surgery, chemotherapy (EP), and radiation therapy, have limited efficacy.^4^ Herein, we excavated a novel potential therapeutic target specifically for NECC. Through cellular communication analysis and functional validation assays, we excitedly noted that DLL3 functioned as a pivotal molecular hub, bridging malignant progression (proliferation and invasion) and immunosuppression (T cell exhaustion and impaired T cell activation) in NECC TIME. The multifaceted tumor-promoting role and widespread expression of DLL3 revealed by IHC staining, together, suggested the potential of DLL3 as a novel therapeutic target for NECC to not only halt tumor progression but also restore immune reaction. To evaluate the effect of DLL3-targeted therapy (AMG757) and its potential in enhancing the frontline chemotherapy regimen (EP) for NECC, effective models for NECC are urgently needed. To date, researchers have not identified a suitable cell line for NECC, or generated animal models. Emerging PDX models, face obstacles due to low success rates and the scarcity of NECC tissue samples. Additionally, even successfully established PDX models ultimately lose the human tumor microenvironment, as murine stroma replaces it during growth.^29^ To address such issues, we established a NECC-derived organoid replicating parental tumor characteristics, and innovatively co-cultured it with TILs to demonstrate the impact of anti-DLL3 on immune cells. Remarkably, the group treated with DLL3-targeted therapy combined with chemotherapy (EP + AMG757) showed significant T cell accumulation, T cell activation, and pronounced T cell-mediated lysis of tumor cells. Inspiringly, AMG 757 has shown manageable safety and promising durability of response in a phase I clinical trial for small cell lung cancer,^30^ strengthening our confidence in its potential as a target for future NECC clinical trials. Together, we pioneeringly established a NECC-derived organoid-TIL co-culture system and proved the efficacy of DLL3-targeted therapy to activate T cells, enhance immunogenic cell death, and improve chemotherapy sensitivity, which provided compelling evidence supporting DLL3-targeted therapy for NECC patients.

This study still has certain limitations. 1) The rarity of NECC presented significant challenges in acquiring sufficient clinical samples for both single-cell analysis and organoid validation. While our current findings provide important preliminary insights into NECC's unique TIME, the limited sample size underscores the need for validation in larger cohorts to strengthen these observations. 2) Given the lack of mature cellular or animal models for NECC, we merely assessed DLL3-targeted therapeutic efficacy in this current study but failed to conduct further *in vivo* studies and mechanistic investigations regarding NPC state transitions and DLL3-associated malignant transformation. 3) Although NECC-derived organoids offer a valuable *in vitro* model system, additional validation is required to determine their capacity to fully recapitulate the spectrum of tumor–immune interactions observed *in vivo*.

In conclusion, NECC is an aggressive histological variant of cervical cancer characterized by “4U” features. To gain a deeper understanding of these characteristics and address the associated challenges, we conducted a series of studies and experimental validations: 1) through scRNA-seq analysis, we identified SYN and INSM1-expressing NPCs with three differentiated states (ENTPD2^+^ IFITM3^+^ edNPCs reshaping TIME, FAM162A^+^ PFKFB3^+^ tdNPCs reprogramming cellular metabolism, and CDKN3^+^ MKI67^+^ ldNPCs over-activating the cell cycle), as the potential malignant origin of NECC, and noted the extensive infiltration of naïve T cells and Tex cells contributing to the immunosuppressive TIME. 2) Based on cellular interaction analysis and functional validation assays, we discovered that DLL3 expressed on NPCs acted as a pivotal molecular hub bridging malignant progression(proliferation and invasion) and immunosuppression (T cell exhaustion and impaired T cell activation) in NECC TIME via interactions with NOTCH2 expressed on NPCs and NOTCH1/2 expressed on TILs. Additionally, IHC staining validated the high and specific expression of DLL3 in NECC, highlighting DLL3 as a promising therapeutic target. 3) We not only innovatively established NECC-derived organoids replicating cellular morphology and neuroendocrine differentiation characteristics of parental tumors, but also co-cultured the organoids with TILs to assess the efficacy of DLL3-targeted therapy (AMG757). Significantly, the combination of AMG757 targeting DLL3 with chemotherapy (EP) induced T cell aggregation and activation, resulting in increased immunogenic cell death and sensitized chemotherapy. This co-culture model provides a robust platform for *in vitro* studies of NECC, and the promising results of DLL3-targeted therapy highlight its potential as a critical therapeutic avenue for NECC patients, offering significant clinical implications.

## CRediT authorship contribution statement

**Qinqin Liu:** Validation, Writing – original draft, Investigation. **Xinyu Qu:** Writing – review & editing, Conceptualization, Formal analysis. **Shuqi Li:** Data curation, Methodology. **Yan Ding:** Software, Methodology. **Tingting Ren:** Methodology, Investigation. **Qi'an Jiang:** Resources, Data curation. **Jingxin Ding:** Formal analysis, Software, Conceptualization. **Keqin Hua:** Supervision, Funding acquisition. **Junjun Qiu:** Funding acquisition, Supervision, Conceptualization.

## Ethics declaration

The current study was approved by the Ethics Committee of Obstetrics and Gynecology Hospital of Fudan University (2022-105), and informed consent was obtained from all participants for sample collection.

## Data availability

The data that support the findings of this study are available from the corresponding author upon reasonable request.

## Funding

This project was supported by funding from the National Natural Science Foundation of China (No. 82472993, 82173188), Medical Innovation Research of Shanghai Science and Technology (China) (No. 22Y31900500), and Shanghai Hospital Development Center (China) (No. SHDC2020CR6009, SHDC2020CR1045B, SHDC22021307, SHDC2020CR4087); Medical Innovation Research of Shanghai Science and Technology (China) (No. 21Y 11906900), the Eastern Talent Plan Project (China), the Simulated RCT study of Shanghai Shenkang Hospital Development Center (China) (No. SHDC2024CRI060), and Huangpu Youth Talent Project.

## Conflict of interests

The authors declare that they have no competing interests.

## References

[1] Sung H., Ferlay J., Siegel R.L. (2021). Global cancer statistics 2020: GLOBOCAN estimates of incidence and mortality worldwide for 36 cancers in 185 countries. CA Cancer J Clin.

[2] Gadducci A., Carinelli S., Aletti G. (2017). Neuroendrocrine tumors of the uterine cervix: a therapeutic challenge for gynecologic oncologists. Gynecol Oncol.

[3] Zhang X., Lv Z., Lou H. (2019). The clinicopathological features and treatment modalities associated with survival of neuroendocrine cervical carcinoma in a Chinese population. BMC Cancer.

[4] Tempfer C.B., Tischoff I., Dogan A. (2018). Neuroendocrine carcinoma of the cervix: a systematic review of the literature. BMC Cancer.

[5] Burzawa J., Gonzales N., Frumovitz M. (2015). Challenges in the diagnosis and management of cervical neuroendocrine carcinoma. Expert Rev Anticancer Ther.

[6] Glaviano A., Lau H.S., Carter L.M. (2025). Harnessing the tumor microenvironment: targeted cancer therapies through modulation of epithelial-mesenchymal transition. J Hematol Oncol.

[7] Binnewies M., Roberts E.W., Kersten K. (2018). Understanding the tumor immune microenvironment (TIME) for effective therapy. Nat Med.

[8] Schultheis A.M., de Bruijn I., Selenica P. (2022). Genomic characterization of small cell carcinomas of the uterine cervix. Mol Oncol.

[9] Xing D., Zheng G., Schoolmeester J.K. (2018). Next-generation sequencing reveals recurrent somatic mutations in small cell neuroendocrine carcinoma of the uterine cervix. Am J Surg Pathol.

[10] Pei X., Xiang L., Chen W. (2021). The next generation sequencing of cancer-related genes in small cell neuroendocrine carcinoma of the cervix. Gynecol Oncol.

[11] Takayanagi D., Hirose S., Kuno I. (2021). Comparative analysis of genetic alterations, HPV-Status, and PD-L1 expression in neuroendocrine carcinomas of the cervix. Cancers (Basel).

[12] Hillman R.T., Cardnell R., Fujimoto J. (2020). Comparative genomics of high grade neuroendocrine carcinoma of the cervix. PLoS One.

[13] Mahdi H., Joehlin-Price A., Elishaev E., Dowlati A., Abbas A. (2021). Genomic analyses of high-grade neuroendocrine gynecological malignancies reveal a unique mutational landscape and therapeutic vulnerabilities. Mol Oncol.

[14] Pan B., Yan S., Yuan L. (2024). Multiomics sequencing and immune microenvironment characteristics define three subtypes of small cell neuroendocrine carcinoma of the cervix. J Pathol.

[15] Ashburner M., Ball C.A., Blake J.A. (2000). Gene ontology: tool for the unification of biology. The gene Ontology Consortium. Nat Genet.

[16] Draghici S., Khatri P., Tarca A.L. (2007). A systems biology approach for pathway level analysis. Genome Res.

[17] Yaari G., Yaari G., Bolen C.R., Thakar J., Kleinstein S.H. (2013). Quantitative set analysis for gene expression: a method to quantify gene set differential expression including gene-gene correlations. Nucleic Acids Res.

[18] Subramanian A., Tamayo P., Mootha V.K. (2005). Gene set enrichment analysis: a knowledge-based approach for interpreting genome-wide expression profiles. Proc Natl Acad Sci U S A.

[19] Vento-Tormo R., Efremova M., Botting R.A. (2018). Single-cell reconstruction of the early maternal-fetal interface in humans. Nature.

[20] Tang Z., Li C., Kang B., Gao G., Li C., Zhang Z. (2017). GEPIA: a web server for cancer and normal gene expression profiling and interactive analyses. Nucleic Acids Res.

[21] Qu X., Wang Y., Jiang Q. (2023). Interactions of indoleamine 2,3-dioxygenase-expressing LAMP3(^+^) dendritic cells with CD4(^+^) regulatory T cells and CD8(^+^) exhausted T cells: synergistically remodeling of the immunosuppressive microenvironment in cervical cancer and therapeutic implications. Cancer Commun.

[22] Lõhmussaar K., Oka R., Espejo Valle-Inclan J. (2021). Patient-derived organoids model cervical tissue dynamics and viral oncogenesis in cervical cancer. Cell Stem Cell.

[23] Chumduri C., Gurumurthy R.K., Berger H., et al. (2021). Opposing Wnt signals regulate cervical squamocolumnar homeostasis and emergence of metaplasia. Nat Cell Biol.

[24] Philip M., Fairchild L., Sun L. (2017). Chromatin states define tumour-specific T cell dysfunction and reprogramming. Nature.

[25] He J., Xiong X., Yang H. (2022). Defined tumor antigen-specific T cells potentiate personalized TCR-T cell therapy and prediction of immunotherapy response. Cell Res.

[26] Allard B., Allard D., Buisseret L., Stagg J. (2020). The adenosine pathway in immuno-oncology. Nat Rev Clin Oncol.

[27] Furuta M., Kikuchi H., Shoji T. (2019). DLL3 regulates the migration and invasion of small cell lung cancer by modulating Snail. Cancer Sci.

[28] Hoyne G.F., Chapman G., Sontani Y., Pursglove S.E., Dunwoodie S.L. (2011). A cell autonomous role for the Notch ligand Delta-like 3 in αβ T-cell development. Immunol Cell Biol.

[29] Yoshida G.J. (2020). Applications of patient-derived tumor xenograft models and tumor organoids. J Hematol Oncol.

[30] Paz-Ares L., Champiat S., Lai W.V. (2023). Tarlatamab, a first-in-class DLL3-Targeted bispecific T-Cell engager, in recurrent small-cell lung cancer: an open-label, phase I study. J Clin Oncol.

