# Supplementary material for: DLL3 serves as a pivotal hub bridging malignant progression and immunosuppression in NECC with therapeutic implications validated via the organoid-TIL co-culture system
Source: Genes Dis. 2025 Jun 30;13(6):101737. doi: 10.1016/j.gendis.2025.101737 (PMC13475434; doi:10.1016/j.gendis.2025.101737)
Supplement: Supplementary file 1 [file mmc1.docx]

**Supporting information**

**Supplementary Table S1**

Clinical characteristics of each sample and numbers of cells sequenced after quality control procedures used for scRNA-seq

| **Sample** | **Pathological Diagnosis** | **Experimental Method** |
| --- | --- | --- |
| NECC_1 | Neuroendocrine carcinoma of the cervix | scRNA-seq + TCR-seq + mIHC |
| NECC_2 | Neuroendocrine carcinoma of the cervix | scRNA-seq + TCR-seq |
| ADC_1 | adenocarcinoma of cervix | scRNA-seq + TCR-seq |
| ADC_2 | adenocarcinoma of cervix | scRNA-seq + TCR-seq |
| SCC_1 | squamous cell carcinoma | scRNA-seq + TCR-seq |
| SCC_2 | squamous cell carcinoma | scRNA-seq + TCR-seq |
| NECC3 | Neuroendocrine carcinoma of the cervix | Establishment of NECC-derived organoid + HE + IHC + mIHC |
| NECC4 | Neuroendocrine carcinoma of the cervix | IHC |
| NECC5 | Neuroendocrine carcinoma of the cervix | IHC |
| NECC6 | Neuroendocrine carcinoma of the cervix | IHC |
| NECC7 | Neuroendocrine carcinoma of the cervix | IHC |
| NECC8 | Neuroendocrine carcinoma of the cervix | IHC |
| NECC9 | Neuroendocrine carcinoma of the cervix | IHC |
| NECC10 | Neuroendocrine carcinoma of the cervix | IHC |
| NECC11 | Neuroendocrine carcinoma of the cervix | IHC |
| NECC12 | Neuroendocrine carcinoma of the cervix | IHC |
| NECC13 | Neuroendocrine carcinoma of the cervix | IHC |
| NECC14 | Neuroendocrine carcinoma of the cervix | IHC |
| NECC15 | Neuroendocrine carcinoma of the cervix | IHC |
| NECC16 | Neuroendocrine carcinoma of the cervix | IHC |
| NECC17 | Neuroendocrine carcinoma of the cervix | IHC |
| ADC_3 | adenocarcinoma of cervix | IHC + mIHC |
| ADC_4 | adenocarcinoma of cervix | IHC |
| ADC_5 | adenocarcinoma of cervix | IHC |
| ADC_6 | adenocarcinoma of cervix | IHC |
| ADC_7 | adenocarcinoma of cervix | IHC |
| ADC_8 | adenocarcinoma of cervix | IHC |
| ADC_9 | adenocarcinoma of cervix | IHC |
| ADC_10 | adenocarcinoma of cervix | IHC |
| ADC_11 | adenocarcinoma of cervix | IHC |
| SCC_3 | squamous cell carcinoma | IHC + mIHC |
| SCC_4 | squamous cell carcinoma | IHC |
| SCC_5 | squamous cell carcinoma | IHC |
| SCC_6 | squamous cell carcinoma | IHC |
| SCC_7 | squamous cell carcinoma | IHC |
| SCC_8 | squamous cell carcinoma | IHC |
| SCC_9 | squamous cell carcinoma | IHC |
| SCC_10 | squamous cell carcinoma | IHC |
| SCC_11 | squamous cell carcinoma | IHC |
| SCC_12 | squamous cell carcinoma | IHC |
| SCC_13 | squamous cell carcinoma | IHC |
| SCC_14 | squamous cell carcinoma | IHC |

**Abbreviations**

NECC, Neuroendocrine carcinoma of the cervix; ADC, adenocarcinomas of the cervix; SCC, squamous cell carcinomas; scRNA-seq, single-cell RNA sequencing; TCR-seq, T cell receptor sequencing; mIHC, multicolor immunohistochemistry; IHC, immunohistochemical staining

**Supplementary Table S2. Antibodies used for flow cytometry analysis, mIHC, and IHC staining.**

| **Antibody** | **Supplier** | **Identifier** | **Experimental Method** | **Fluorescence** |
| --- | --- | --- | --- | --- |
| L/D | BD Biosciences | 564406 | Cytometry analysis | FVS510 |
| Human anti-CD45 | BD Biosciences | 557833 | Cytometry analysis | APC-CY7 |
| Human anti-CD3 | BD Biosciences | 555339 | Cytometry analysis | FITC |
| Human anti-CD4 | BD Biosciences | 562658 | Cytometry analysis | BV605 |
| Human anti-Perforin | BD Biosciences | 563393 | Cytometry analysis | BV421 |
| Human anti-CD107a | BD Biosciences | 566558 | Cytometry analysis | BB700 |
| Human anti-CD137 | BioLegend | 309827 | Cytometry analysis | BV650 |
| Human anti-GZMB | BioLegend | 515406 | Cytometry analysis | AF647 |
| Human anti-PDCD1 | BioLegend | 621612 | Cytometry analysis | FITC |
| Human anti-TIM3 | BioLegend | 345032 | Cytometry analysis | BV786 |
| Anti- PECAM1 | Abcam | ab182981 | mIHC staining |  |
| Anti-CD38 | Abcam | ab108403 | mIHC staining |  |
| Anti-CD8 | CST | #70306S | mIHC staining |  |
| Anti-CD138 | Abcam | ab128936 | mIHC staining |  |
| Anti-PDCD1 | CST | #86163S | mIHC staining |  |
| Anti-NOTCH1 | Abcam | ab52627 | mIHC staining |  |
| Anti-NOTCH2 | CST | 5732T | mIHC staining |  |
| Anti-DLL3 | Abcam | ab229902 | mIHC staining; IHC staining |  |
| Anti-SYN | Abcam | ab32127 | mIHC staining; IHC staining |  |
| Anti-INSM1 | Abcam | ab305104 | IHC staining |  |
| β-actin monoclonal antibody | Proteintech | No. 66009-1-Ig | Western blot |  |
| DLL3 Polyclonal antibody | Proteintech | No. 25535-1-AP | Western blot |  |

**Supplementary Figure S1.** **Features, classification and functions of the epithelial cells and NPCs in CC TIME**

(A) Representative IHC staining for SYN and INSM1 in samples of NECC, SCC, and ADC. Scale bars: 200 μm and 50 μm.

(B) Violin plot showing CNV scores of endothelial cells, epithelial cells, myeloid cells and NPCs derived from NECC, ADC, and SCC.

(C)A UMAP plot exhibiting 12 subclusters generated via epithelial cells reclustering.

(D) A UMAP plot showing malignant and nonmalignant epithelial cells determined using inferCNV analysis.

(E) Bar plot showing the enriched pathways of up gene expression in malignant vs. nonmalignant epithelial cells derived from NECC (left), SCC (middle) and ADC (right).

**Abbreviations**

NPCs, neuronal progenitor cells; CC, cervical cancer; TIME, tumor immune microenvironment; IHC, immunohistochemistry; NECC, neuroendocrine carcinoma of the cervix; SCC, squamous cell carcinomas; ADC, adenocarcinomas of the cervix; CNV, copy number variation

**Supplementary Figure S2. Three differentiated states of NPCs.**

Trajectory analysis of each cluster of NPCs derived from NECC.

**Abbreviations**

NPCs, neuronal progenitor cells; NECC, neuroendocrine carcinoma of the cervix

**Supplementary Figure S3. The subclusters of CD4+ T cells, CD8+ T cells and B cells from CC samples.**

(A-B) The UMAP plot showing the 9 subclusters generated from CD4+ T cells (A) reclustering and 9 subclusters generated from CD8+ T cells (B) reclustering.

(C-E) Heatmap of marker gene expression of the 9 subclusters of CD4+ T cells (C), 9 subclusters generated from CD8+ T cells (D), and 10 subclusters of B cells (E) reclustering.

**Abbreviations**

CC, cervical cancer

**Supplementary Figure S4. The validation of intercellular interactions and the expression of DLL3 in NECC, ADC, and SCC samples.**

(A) Representative images of mIHC staining for lymphoid aggregates, and the co-localization of CD8+PDCD1+PECAM1+ T cells (blue arrow) and CD38+CD138+ plasma cells (white arrow) in lymphoid aggregates of NECC. The orange, green, grey, red and yellow arrows indicate cells in NECC tissue samples with positive expression of the CD8, PDCD1, PECAM1, CD38, and CD138 proteins, respectively. Scale bars, 100 μm and 20 μm for the top and bottom panels, respectively.

(B) Representative images of mIHC staining for showing the co-localization of DLL3+ tumor cells and NOTCH2+SYN+ tumor cells in NECC. The green, grey, and cyan-blue arrows indicate cells in NECC tissue samples with positive expression of the SYN, DLL3, and NOTCH2 proteins, respectively. Scale bars, 100 μm and 20 μm for the top and bottom panels, respectively.

(C) Representative images of mIHC staining illustrating the presence of SYN (green), NOTCH1 (orange), NOTCH2 (cyan-blue), CD8 (yellow), CD38 (red), and DAPI (blue). The scale bar, 100 μM. Notably, DLL3 tumor cells were not observed in ADC/SCC.

(D) qRT-PCR results showed that DLL3 was overexpressed in Hela-DLL3 OE (upper) and successfully knocked down in NCI-H82-KD (lower) (n ≥ 3 per group).

(E) Western blot results demonstrated the expression of DLL3 protein in Hela-NC, Hela-DLL3 OE, NCI-H82-NC, and NCI-H82-DLL3 KD cells, with significant upregulation in Hela-DLL3 OE and downregulation in NCI-H82-DLL3 KD compared to their respective controls. (n ≥ 3 per group).

(F) PDCD1 inhibitor Toripalimab did not significantly alter tumor cell mortality rates in either HeLa NC versus HeLa-DLL3 OE (left, P = 0.0543) or NCI-H82 NC versus NCI-H82-DLL3 KD (right, P = 0.0922) comparisons.

**Abbreviations**

NECC, neuroendocrine carcinoma of the cervix; ADC, adenocarcinomas of the cervix; SCC, squamous cell carcinomas; mIHC, multiplex immunohistochemistry; IHC, immunohistochemistry; NC, negative control

*, P < 0.05; **, P < 0.01; ***, p <0.001; ****, P<0.0001. ns, not significant; Error bar: mean value with 95% CI

**Supplementary Figure S5.** Genomic and Transcriptomic Concordance between NECC and NECC-Derived Organoids

(A)Heatmap showed the similar gene expression profile between NECC and NECC-derived organoid.

(B)Bar graphs displayed the different contributions of SNV in NECC and NECC-derived organoid.

(C)The heatmap showed the top50 gene mutation type in NECC and NECC-derived organoid.

**Abbreviations**

NECC, neuroendocrine carcinoma of the cervix; SNV, Single Nucleotide Variant

**Supplementary Figure S6. Flow cytometry gating strategy for identifying CD8+ T cells and cytotoxic effectors CD8+T cells.**

First, cells were positioned using forward and side scatter regions, then doublets were excluded by gating based on forward scatter and height. Live cells were gated using L/D dye, and leukocytes were gated through CD45+ cells. Cells within the leukocyte gate were further gated based on CD3+CD8+ T cells. The positive gating for cytotoxic effectors (CD137, CD107a, Perforin, GZMB, PDCD1, TIM3) is shown in the lower part of the figure.
